# Supplementary material for: Chronic Variable Stress May Induce Apoptosis in the Testis and Epididymal Sperm of Young Male Rats
Source: Biology (Basel). 2025 Jun 12;14(6):690. doi: 10.3390/biology14060690 (PMC12189084; doi:10.3390/biology14060690)
Supplement: Supplementary file 1 [file biology-14-00690-s001.zip › biology-3651179-supplementary.pdf]

Supplementary material

Figures: details of western blot membranes in epididymis sperm

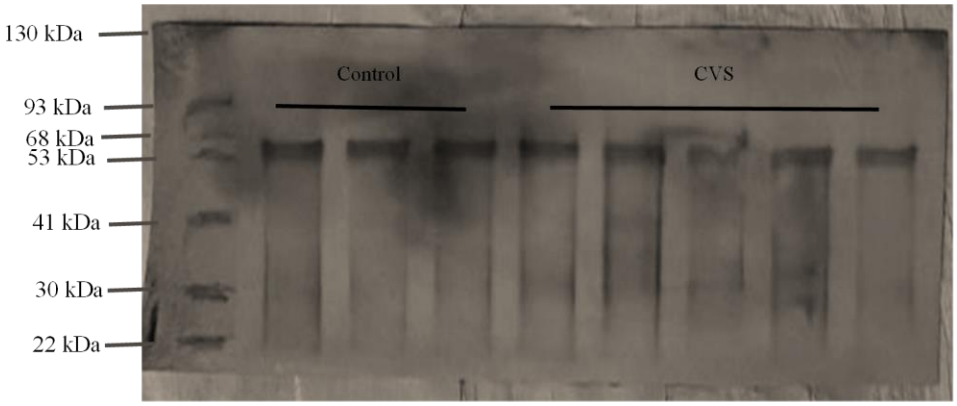

Figure S1-1. Western Blot membrane of Akt protein (56 kDa) detected with anti-Akt (Santa Cruz Biotechnology, Inc.USA; 1:1500). SDS page was transferred to nitrocellulose membrane (0.2 µm pore size; Bio-Rad) by semidry electroblotting (100 V, 2 hours). Membrane was incubated with an anti-rabbit secondary antibody (PROMEGA, Madison, WI. USA; 1:1000). The detection of immunoblotting bands was measured using BCIP®/NBT solution (B6404-Sigma Aldrich, St. Louis, MO, USA). Weight marker (kDa) used: prestained protein marker with molecular weights ranging from 9kDa to 170kDa. The immunoreactive bands were analyzed by Image Lab Software 6.0.1. (Bio-Rad Laboratories, Inc). Blot images were converted to grayscale with Adobe Photoshop CC software (Adobe Photoshop Version: 14.0). Control: control group; CVS: chronic variable group.

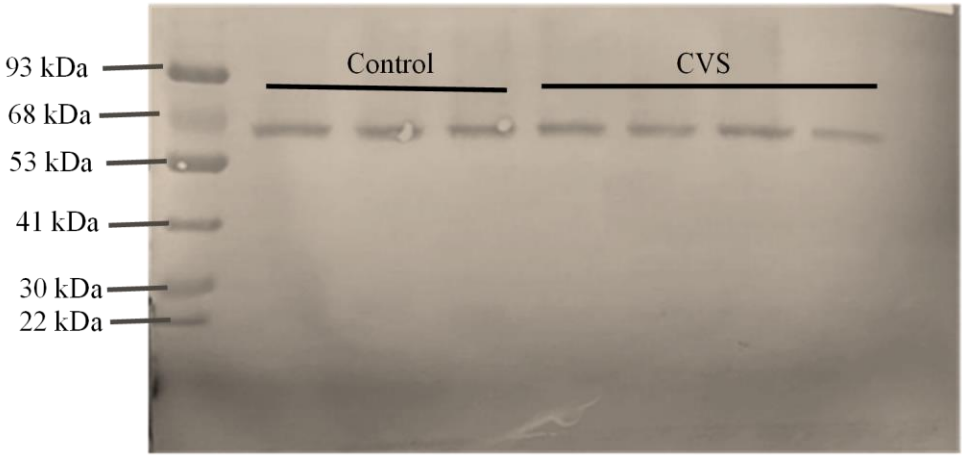

Figure S1-2. Western Blot membrane of AP-2α protein (55 kDa) detected with AP-2α antibody (Santa Cruz Biotechnology, Inc.USA; 1:1500). SDS page was transferred to nitrocellulose membrane (0.2 µm pore size; Bio-Rad) by semidry electroblotting (100 V, 2 hours). Membrane was incubated with goat anti-mouse secondary antibody (Santa Cruz Biotechnology, Inc; 1:1000). The detection of immunoblotting bands was measured using BCIP®/NBT solution (B6404-Sigma Aldrich, St. Louis, MO, USA). Weight marker (kDa) used: prestained protein marker with molecular weights ranging from 9kDa to 170kDa. The immunoreactive bands were analyzed by Image Lab Software 6.0.1. (Bio-Rad Laboratories, Inc). Blot images were converted to grayscale with Adobe Photoshop CC software (Adobe Photoshop Version: 14.0). Control: control group; CVS: chronic variable group.

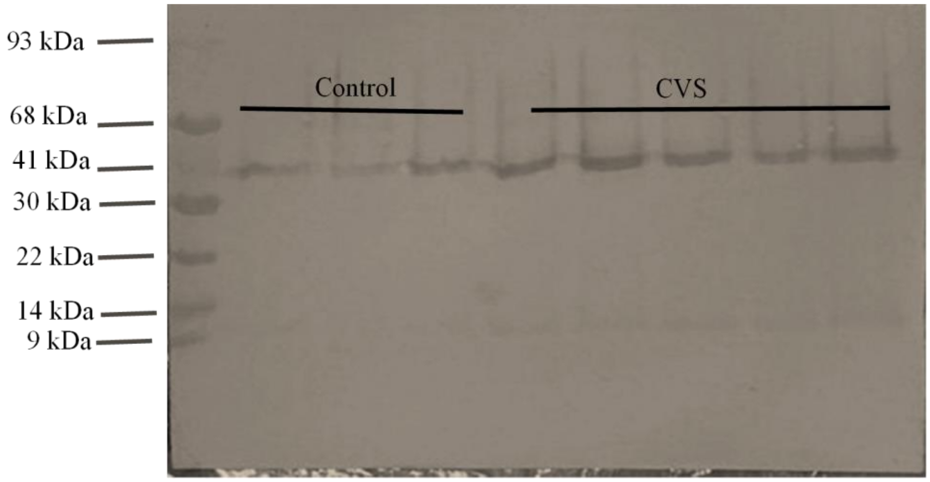

Figure S1-3. Western Blot membrane of FAS protein (36 kDa) detected with FAS antibody (Santa Cruz Biotechnology, Inc.USA; 1:1500). SDS page was transferred to nitrocellulose membrane (0.2 µm pore size; Bio-Rad) by semidry electroblotting (100 V, 2 hours). Membrane was incubated with goat anti-mouse secondary antibody (Santa Cruz Biotechnology, Inc; 1:1000). The detection of immunoblotting bands was measured using BCIP®/NBT solution (B6404-Sigma Aldrich, St. Louis, MO, USA). Weight marker (kDa) used: prestained protein marker with molecular weights ranging from 9kDa to 170kDa. The immunoreactive bands were analyzed by Image Lab Software 6.0.1. (Bio-Rad Laboratories, Inc). Blot images were converted to grayscale with Adobe Photoshop CC software (Adobe Photoshop Version: 14.0). Control: control group; CVS: chronic variable group.

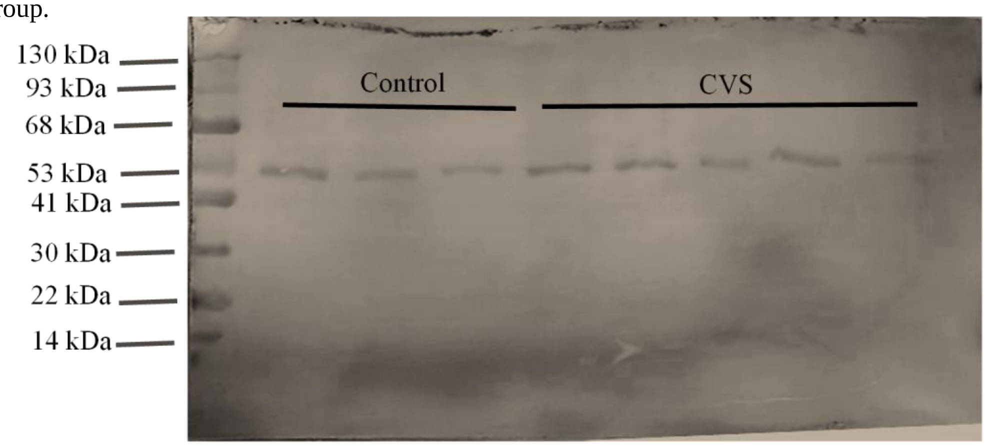

Figure S1-4. Western Blot membrane of PPARγ protein (56 kDa) detected with PPARγ antibody (Santa Cruz Biotechnology, Inc.USA; 1:1000). SDS page was transferred to nitrocellulose membrane (0.2 µm pore size; Bio-Rad) by semidry electroblotting (100 V, 2 hours). Membrane was incubated with goat anti-mouse secondary antibody (Santa Cruz Biotechnology, Inc; 1:1000). The detection of immunoblotting bands was measured using BCIP®/NBT solution (B6404-Sigma Aldrich, St. Louis, MO, USA). Weight marker (kDa) used: prestained protein marker with molecular weights ranging from 9kDa to 170kDa. The immunoreactive bands were analyzed by Image Lab Software 6.0.1. (Bio-Rad Laboratories, Inc). Blot images were converted to grayscale with Adobe Photoshop CC software (Adobe Photoshop Version: 14.0). Control: control group; CVS: chronic variable group.

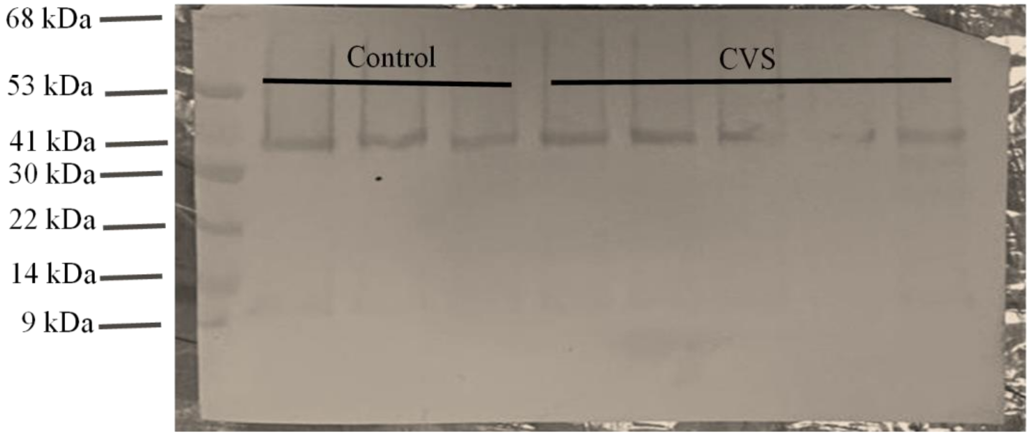

Figure S1-5. Western Blot membrane of C/EBP-β protein (43 kDa) detected with C/EBP-β antibody (Santa Cruz Biotechnology, Inc.USA; 1:1000). SDS page was transferred to nitrocellulose membrane (0.2 μm pore size; Bio-Rad) by semidry electroblotting (100 V, 2 hours). Membrane was incubated with goat anti-mouse secondary antibody (Santa Cruz Biotechnology, Inc; 1:1000). The detection of immunoblotting bands was measured using BCIP®/NBT solution (B6404-Sigma Aldrich, St. Louis, MO, USA). Weight marker (kDa) used: HyperPAGE II prestained protein marker with molecular weights ranging from 9kDa to 170kDa. The immunoreactive bands were analyzed by Image Lab Software 6.0.1. (Bio-Rad Laboratories, Inc). Blot images were converted to grayscale with Adobe Photoshop CC software (Adobe Photoshop Version: 14.0). Control: control group; CVS: chronic variable group.

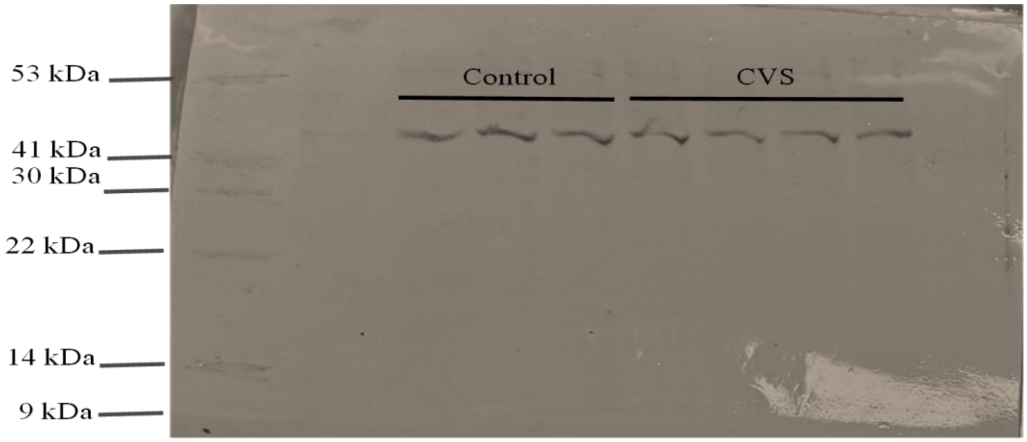

Figure S1-6. Western Blot membrane of β-actin protein (43 kDa) detected with -actin (c4) SC-47778 antibody from Santa Cruz Biotechnology, Inc.USA; 1:1000). SDS page was transferred to nitrocellulose membrane (0.2 μm pore size; Bio-Rad) by semidry electroblotting (100 V, 2 hours). Membrane was incubated with goat anti-mouse secondary antibody (Santa Cruz Biotechnology, Inc; 1:1000). Weight marker (kDa) used: protein marker with molecular weights ranging from 9kDa to 170kDa. The immunoreactive bands were analyzed by Image Lab Software 6.0.1. (Bio-Rad Laboratories, Inc). Blot images were converted to grayscale with Adobe Photoshop CC software (Adobe Photoshop Version: 14.0). Control: control group; CVS: chronic variable group.

Figures: details of western blot membranes in complete testicles

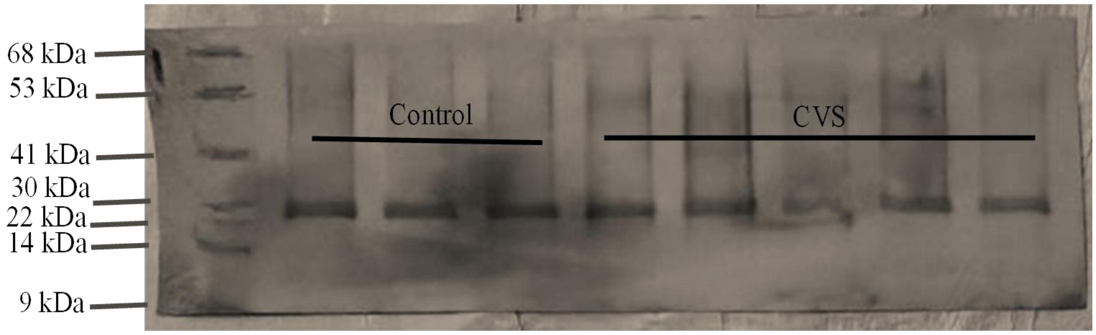

Figure S2-1. Western Blot membrane of Akt protein (56 kDa) detected with anti-Akt (Santa Cruz Biotechnology, Inc.USA; 1:1500). SDS page was transferred to nitrocellulose membrane (0.2 µm pore size; Bio-Rad) by semidry electroblotting (100 V, 2 hours). Membrane was incubated with an anti-rabbit secondary antibody (PROMEGA, Madison, WI. USA; 1:1000). The detection of immunoblotting bands was measured using BCIP®/NBT solution (B6404-Sigma Aldrich, St. Louis, MO, USA). Weight marker (kDa) used: prestained protein marker with molecular weights ranging from 9kDa to 170kDa. The immunoreactive bands were analyzed by Image Lab Software 6.0.1. (Bio-Rad Laboratories, Inc). Blot images were converted to grayscale with Adobe Photoshop CC software (Adobe Photoshop Version: 14.0). Control: control group; CVS: chronic variable group.

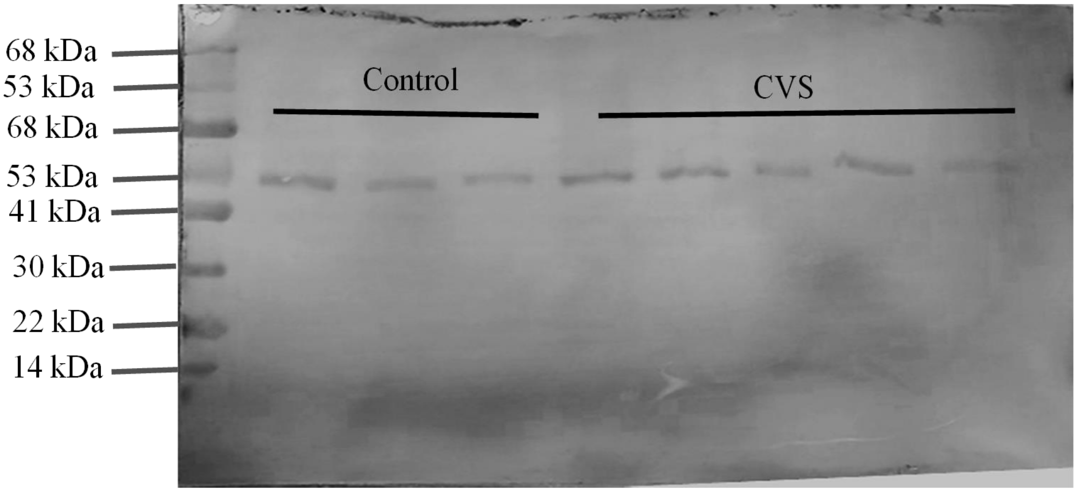

Figure S2-2. Western Blot membrane of AP-2α protein (55 kDa) detected with AP-2α antibody (Santa Cruz Biotechnology, Inc.USA; 1:1500). SDS page was transferred to nitrocellulose membrane (0.2 µm pore size; Bio-Rad) by semidry electroblotting (100 V, 2 hours). Membrane was incubated with goat anti-mouse secondary antibody (Santa Cruz Biotechnology, Inc; 1:1000). The detection of immunoblotting bands was measured using BCIP®/NBT solution (B6404-Sigma Aldrich, St. Louis, MO, USA). and chemiluminescence (ECL) was used to detect signals under the gel doc apparatus (Bio-Rad laboratories, Inc). Weight marker (kDa) used: prestained protein marker with molecular weights ranging from 9kDa to 170kDa. The immunoreactive bands were analyzed by Image Lab Software 6.0.1. (Bio-Rad Laboratories, Inc). Blot images were converted to grayscale with Adobe Photoshop CC software (Adobe Photoshop Version: 14.0). Control: control group; CVS: chronic variable group.

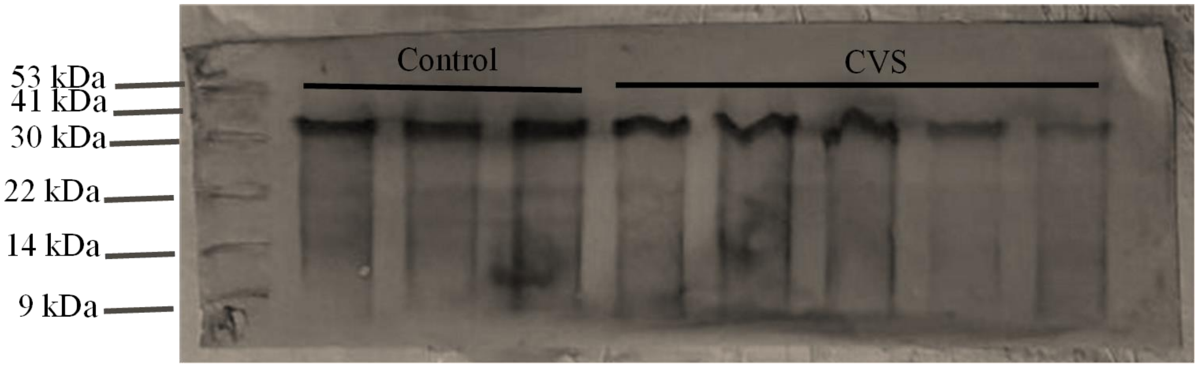

Figure S2-3. Western Blot membrane of FAS protein (36 kDa) detected with FAS antibody (Santa Cruz Biotechnology, Inc.USA; 1:1500). SDS page was transferred to nitrocellulose membrane (0.2 µm pore size; Bio-Rad) by semidry electroblotting (100 V, 2 hours). Membrane was incubated with goat anti-mouse secondary antibody (Santa Cruz Biotechnology, Inc; 1:1000). The detection of immunoblotting bands was measured using BCIP®/NBT solution (B6404-Sigma Aldrich, St. Louis, MO, USA). Weight marker (kDa) used: marker with molecular weights ranging from 9 kDa to 170 kDa. The immunoreactive bands were analyzed by Image Lab Software 6.0.1. (Bio-Rad Laboratories, Inc). Blot images were converted to grayscale with Adobe Photoshop CC software (Adobe Photoshop Version: 14.0). Control: control group; CVS: chronic variable group.

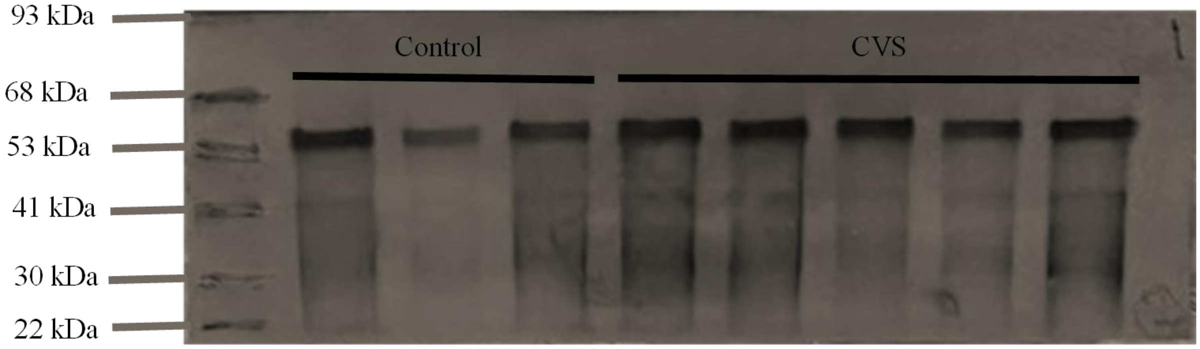

Figure S2-4. Western Blot membrane of PPARγ protein (56 kDa) detected with PPARγ antibody (Santa Cruz Biotechnology, Inc.USA; 1:1000). SDS page was transferred to nitrocellulose membrane (0.2 µm pore size; Bio-Rad) by semidry electroblotting (100 V, 2 hours). Membrane was incubated with goat anti-mouse secondary antibody (Santa Cruz Biotechnology, Inc; 1:1000). The detection of immunoblotting bands was measured using BCIP®/NBT solution (B6404-Sigma Aldrich, St. Louis, MO, USA). Weight marker (kDa) used: prestained protein marker with molecular weights ranging from 9 kDa to 170 kDa. The immunoreactive bands were analyzed by Image Lab Software 6.0.1. (Bio-Rad Laboratories, Inc). Blot images were converted to grayscale with Adobe Photoshop CC software (Adobe Photoshop Version: 14.0). Control: control group; CVS: chronic variable group.

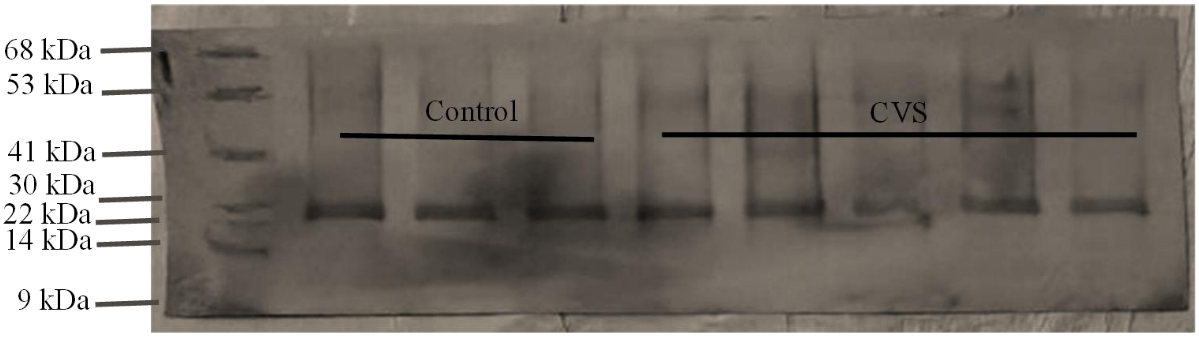

Figure S2-5. Western Blot membrane of C/EBP-β protein (43 kDa) detected with C/EBP-β antibody (Santa Cruz Biotechnology, Inc.USA; 1:1000). SDS page was transferred to nitrocellulose membrane (0.2 µm pore size; Bio-Rad) by semidry electroblotting (100 V, 2 hours). Membrane was incubated with goat anti-mouse secondary antibody (Santa Cruz Biotechnology, Inc; 1:1000). The detection of immunoblotting bands was measured using BCIP®/NBT solution (B6404-Sigma Aldrich, St. Louis, MO, USA). Weight marker (kDa) used: prestained protein marker with molecular weights ranging from 9kDa to 170kDa. The immunoreactive bands were analyzed by Image Lab Software 6.0.1. (Bio-Rad Laboratories, Inc). Blot images were converted to grayscale with Adobe Photoshop CC software (Adobe Photoshop Version: 14.0). Control: control group; CVS: chronic variable group.

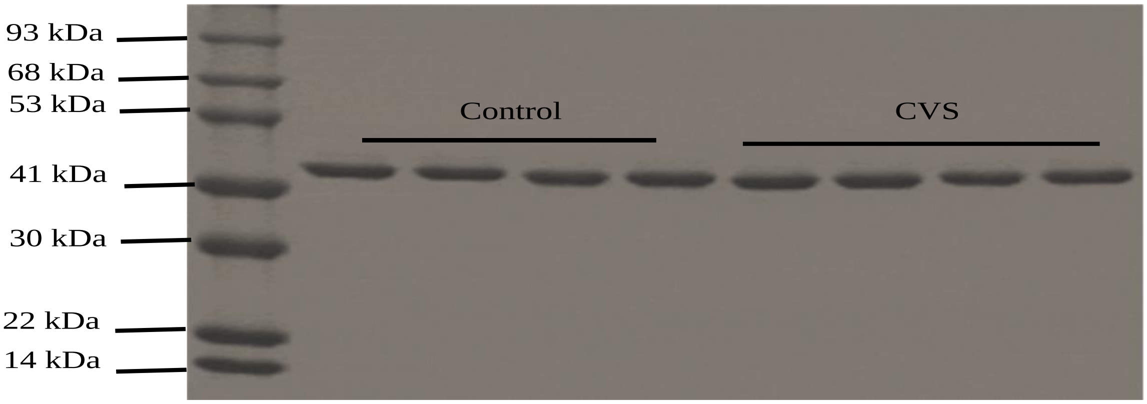

Figure S2-6. Western Blot membrane of β-actin protein (43 kDa) detected with B-actin (c4) SC-47778 antibody from Santa Cruz Biotechnology, Inc.USA; 1:1000). SDS page was transferred to nitrocellulose membrane (0.2 µm pore size; Bio-Rad) by semidry electroblotting (100 V, 2 hours). Membrane was incubated with goat anti-mouse secondary antibody (Santa Cruz Biotechnology, Inc; 1:1000). Weight marker (kDa) used: protein marker with molecular weights ranging from 9kDa to 170kDa. The immunoreactive bands were analyzed by Image Lab Software 6.0.1. (Bio-Rad Laboratories, Inc). Blot images were converted to grayscale with Adobe Photoshop CC software (Adobe Photoshop Version: 14.0). Control: control group; CVS: chronic variable group.
